# Social Communication of Transportation: A Bridge Model Connecting Tourism Destination and Psychological Perception

**DOI:** 10.3389/fpsyg.2021.823884

**Published:** 2022-01-31

**Authors:** Ligang Zhang, Xingrong Wang, Yi Li, Yan Zhu, Feng Wei, Shaoqiong Zhao

**Affiliations:** ^1^School of Journalism, Fudan University, Shanghai, China; ^2^School of Literature and Law, East China University of Technology, Nanchang, China; ^3^College of Economics and Management, East China Jiaotong University, Nanchang, China; ^4^School of Economics and Management, Tsinghua University, Beijing, China; ^5^Institute of Internet Industry, Tsinghua University, Beijing, China; ^6^School of Business and Economics, State University of New York Plattsburgh, Plattsburgh, NY, United States

**Keywords:** social communication, tourism transportation route, point-line link, tourist psychological perception, tourist flow

## Abstract

As it is essential to explore the influence of social communications on transportation routes in tourism, this article aims to examine the impacts of social communications on transportation routes in the field of tourism and to further explore the relationship between tourism destinations and their psychological perceptions. In terms of links between different tourism destinations in space and time dimensions, our empirical analysis draws the following conclusions: (1) the behavior of tourist flow is a mediating variable on the links between tourist psychological perceptions and tour routes; (2) three modes of point-line interaction are presented in the space and time of tourism destinations; and (3) the scenic city’s location, name, and features are important to tourists’ psychological perceptions.

## Introduction

The *Theory of Transportation* means a movement from one place to another, and includes a deeper analysis of the various social functions of transportation (Cooley, 1894); and the research carefully discusses the social communication significance and develops a theory of transportation that examines severally its relations to various social institutions (Cooley, 1894). In the realm of tourism, academia has researched the vital implications of transportation for tourism in the early time and concluded that transportation exists as a cross-domain behavior of tourists to get rid of the spatial constraints posed by geographical locations, which can meet their demands such as the needs for entertainment, consumption, experience, and learning (Ho et al., 2020) and facilitate the effective connection between points of tourist psychological perception. Specifically, the research on the connection of transportation covers a wide range of subjects, including the research on leisure and mobility-oriented use of railway transportation (Li et al., 2021), bus competitiveness based on a comparative assessment of the experience (Altieri et al., 2021), and the impact imposed by the accessibility of air transport on the development of tourism (Papatheodorou, 2021). Previous studies have carried out numerous attempts on the regularity of impact imposed by the special incident of the COVID-19 pandemic, and elaborated on the new situation of COVID-19 with the current measures of pandemic prevention and control, in addition to the resource planning related to the demand preferences of tourists (Florido-Benitez, 2021). Against the backdrop of the era, tour transportation has become a hot-spot issue for varying sorts of relevant studies. In-depth exploration has been made to help plan the layout of tour routes, whereas the macroscopic law of tour routes (*line*) has increasingly drawn interest from academia.

Based on the above views, tourist psychological perception (*point*) and tour route (*line*) have been further summarized and applied in various studies, such as the research on spatial characteristics of tourist psychology and traffic flow in mountain tourism (Konda and Nakamura, 2020), the research of tourist psychological perception on traffic condition during the selection of tourism destinations (Chen et al., 2020), and the research on the relationship between tourism value perceived psychologically and traffic demand, in addition to the requirements for layout planning of tour routes (Liu and Zhang, 2020). Therefore, the tourist psychological perception could determine the planning of tour routes, whereas the planning of tour routes will also impose an impact on the tourist psychological perception in turn, which further influences the cognitions and sentiments of tourists. While elaborating on the relationship between the two factors, previous studies tend to focus on the direct connection of A→B, which seems to be lopsided. Moreover, a paradox seems to exist between tourism and transportation, and there could be a potential impact of the tourism economy on transportation management (Scuttari et al., 2019). In other words, the tourism behavior of the tourist themselves is either a positive or a negative choice of consumption. This perspective has presented a more reasonable intermediary variable, namely, the behavior of tourist flow, existing between tourist psychological perception and tour routes.

The population flow refers to a geographical phenomenon caused by tourist activities from one place to another. As evidenced from numerous studies, such phenomena as population flow and migration can be interpreted by Push-pull Theory. Specifically, the internal thrust serves as a physical expression of tourism motivation which is generally recognized by academia and constitutes a driving force caused by psychological imbalance or tension. In essence, it is deemed as the central value generated by tourist behavior that leads to human flow due to tourists’ seeking mental novelty, and getting away from routine life (Han et al., 2021). By leveraging the internal pushing force, researchers can systematically elaborate the causes of tourism behavior, and the objective of the tourism experience the tourists desire to obtain. From the academic perspective of the external pulling force, it is represented as the attraction of landscape attributes; such attributes are generally embodied by the cultural ambiance, accommodation, and transportation (Lee and Han, 2020). Based on these analyses, academia has proposed the attribution model of loyalty to the tourism destination, and it is recognized that the tourists’ perception of the external pulling force, which is derived from the destination, is generally embodied by the factors such as safety, expression of hospitality, and features of scenic areas (Suhartanto et al., 2021). The application of the theories on both pushing and pulling has constantly been perceived as separate from each other, whereas the respective research has been carried out on the regularity of mental satisfaction and attraction of the destination. However, it shall be noted that the one-way understanding of a specific aspect would be a lopsided perception. In general, while the internal pushing force promotes people to engage in tourism activities, the external pulling force derived from tourism destinations will also work in tandem and lead to the chain reaction of individual mentality and behavior. Such regularity has further revealed the potential correlation between tourist psychological perception and tour routes. Therefore, this study aims to enrich the literature of tourist psychological perception, tour destination, and the behavior of tourist flow in the social communication perspective and further consolidate the research foundation.

Tourist psychological perception constitutes a key factor during the development of tourism, whereas tour transportation serves as the prerequisite for the tourism sector. The *status quo* of the performance of both factors could better reflect the orientation of the tourism development at present. At the critical juncture where the tourism economy is facing a global crisis, it has become a topic worth pondering over in this era which concerns how to apply the tour route (*line*) and how to connect it with the tourist psychological perception (*point*). Based upon the above analysis, the tourism destinations in Jiangxi Province are chosen as the subject of this research to explore the mechanism of the relationship between point and line through an empirical analysis. Subsequently, the keywords on the tourist psychological perception for national 5A-level scenic areas are extracted by grasping, sorting, and analysis of online traveling posts. Integrated with the emotional response, the psychological picture on the tourist perception is illustrated for the space of tourism destinations. Furthermore, based on the latest map on the geographical distribution of arterial highways and express railways, the effective mechanism of the connected relationship between point and line is illustrated through observation. Therefore, the study has come to the conclusions on the logic of point-line in the space of tourism destinations and expects to be of great significance for the subsequent exploration of the developmental law of tourism and transportation.

## Literature Review and Research Hypothesis

This study aims to explore the relationship among tourist psychological perception, tourism destination, and the behavior of tourist flow from the social communication perspective. Previous studies on social communication of transportation combined cultural anthropology and human geography address the physical space influences on the social culture structure, e.g., traffic roads and routes can rebuild the tourist’s transportation flow and shift the tourism culture into the psychological perception in terms of space, time, and social consciousness. Therefore, it is necessary to outbreak the single recognition of geographic space and explore a multi-dimensional perspective to interpret the social communication process and extend individual’s psychological perception into the social structure change, for instance, the intensive geographic routes accelerate the flowing directions and scales of tourists, which produces more opportunities and social communications to bridge the tourism destination and the tourists’ psychological perception.

### Tourist Psychological Perception

Tourism refers to the procedure of relieving mental pressure and adjusting the overall status toward the comfort zone (Stronza et al., 2019). It is an activity that newly emerged after the advancement of human society to a certain extent, which is of self-evident industrial value. According to the definition of the United Nations World Tourism Organisation (2019), tourism destination refers to “*physical spaces with or without administrative and/or analytical boundaries in which a visitor can spend an overnight*”. It is the unit of product and service, and activity and experience along the tourism value chain, and a basic cluster of analysis of tourism. As a result, tourism has constantly drawn interest from the global academia and the tourist psychological perception constitutes the focus of academic research particularly. Judging from numerous studies, practical activities are generally guided by tourist emotions. From the perspective of modern psychology, the individual considers as a system of transferring and processing information. Considering the features of varying matters, the information can be coded and stored in consciousness before being effectively expressed through sound, shape, and text; such mode of experience is referred to as perceptual psychology (Skavronskaya et al., 2017). Originated from the emerging theory of cognitive psychology in the mid-1950s, perceptual psychology has gradually become a separate subject of systematic research as the role of cognitive psychology is increasingly recognized by scholars with a systematic concept (Goldhaber, 2021). Perceptual psychology constitutes the abstract expression of human emotion, which is subject to the influence of space and time. Specifically, during the investigative research conducted by scholars from Fairmount University, it is verified for the first time that the geographical space could impose a positive impact on the changes to human emotion (Fridgen, 1984), thus pioneering the research on the relationship between environmental psychology and tourism. Subsequently, with the gradual advancement made in such research, the scope of perceptual psychology is constantly extended to form a new hot spot for studies on tourism at present.

In general, studies on tourist psychological perception cover a wide scope of topics. For instance, Wei and Dai (2019) explored the attribution of in-depth perception of social power in tourism destinations from the perspective of tourism gaze, which has elaborated on the mutual relationship among varying tourism subjects, and it is found that the host of destination is entitled to greater rights of ensuring that tourists are willing to accept arrangement and control. The forecasted perceived risks of destination, which have taken a holistic view on the relationship among the image of the destination, satisfaction of tourists, and risks, whereas analysis is carried out on the impact imposed by risks and safety on the tourism destinations. It has been widely recognized in the new era, covering such topics as the perception of risks related to health, economy, equipment, society, time, and opportunity (Jonas et al., 2011; Papilaya, 2018; Xie et al., 2020). The environmental perception of tourist experience has been studied, which focuses on the level of satisfaction of tourists’ demands related to the environment; He et al. (2018) has elaborated on the implications and value of tourists’ environmental responsibilities and commitment. In addition, there was research on the recognition of local image, which has elaborated on the reputation of the tourism destination itself at the micro-level (Volgger et al., 2021). At a higher level, the research is conducted concerning the perception of a nation and certain scholars found that the perception of tourism destinations is related to the shaping of national image to a certain extent (Dedeoglu, 2019). Therefore, it shall be noted that regardless of the content perceived by tourists, the tourist psychological perception is originated from the mentality of tourists in essence, which is an organic component of life experience capable of triggering emotions and memories, thereby forming a collection of point distribution in time and space (Pearce, 2020).

As evidenced by numerous previous studies, the tourist psychological perception is the point that drives the development of tourism destinations, which can form a cohesive force (Kim et al., 2020) and facilitate the sustainable development of the tourism sector. The study is of self-evident significance and worth exploring further as the ways of connecting the points that are manifested in varying aspects of perceptual psychology, the distribution of virtual spatial points, the ways of connecting physical space during the high-quality development of the tourism sector at present, and the implementation of further design and research.

### Tour Routes

Transportation serves as an effective carrier of the life trajectory and is a basic element of tourism activities. In terms of research on tour transportation, systematic studies have been carried out on the optimization and computation, which primarily adopt the practical analysis on the planning of tour routes (Wu et al., 2017). Specifically, previous research focused on the clustering center motive iteration search, including precise interesting tourist sight data mining, and heterogeneous tourism data, and sequential pattern mining (Bin et al., 2019a). Regardless of the types of computational methods, the research on the calculation and planning of tour routes centers around tourist preferences and interest fields (Xiao et al., 2018). Even in terms of the analysis on the trend of tourist flow or the maximization of experience utility, the study essentially focuses on the planning and attraction of tourist behaviors (Bin et al., 2019b), which is an extended-expression of the attention from the sociological perspective.

From the perspective of academia, a close relationship exists between sociology and psychology, whereas the behavior is deemed to be induced by mentality or sentiment. Therefore, on one hand, the planning of tour transportation-oriented toward tourists shall meet the tourists’ psychological needs (Lee and Abrahams, 2018; Zheng and Liao, 2019), consistent with the design of railway networks in the macro level of tour planning, and abide by the changing law of the mentality of tourist experience (Li et al., 2020). On the other hand, the planning of tour routes shall focus on the rich architectural, cultural, and scenic values and interpret them based on the interests of tourists (Barzegar et al., 2019). Although few scholars have proposed the opinions that the planning of tour routes shall focus on the psychological features of tourists, it is an integral part of almost all relevant explorations. While the tour transportation exists at the physical level and the tourist perception exists at the psychological level separately, the two factors can virtually have a strong correlation with each other. For instance, tour transportation helps enhance satisfaction from the aspect of tourist psychological perception. Further studies can be carried out on the regularity behind the line of tour transportation and the point of tourist perception on the same level. Therefore, we put forward the first hypothesis:

*H1: The optimization of tour routes can impose a positive impact on the improvement of tourists’ psychology perception.*

### Tourist Flow

Among the studies related to the tourist psychological perception and tour transportation, tourist flow has been an increasingly important topic in both academia and practice. Such behaviors are not only regarded as the outcome of tourist perception of their psychological needs but also the contributing factors to build the tour transportation, which exists as the trajectory of space and time for tourists to meet their needs of tourism (Davis et al., 2018). According to the analysis, the behavior of tourist flow is subject to the influence of two major factors, the subjective psychological factor, and the objective environmental factor. These two factors are correlated with each other, thereby forming a distinct pattern of behavior on tourist flow. However, the integrated studies on the two factors are not common in current research. Instead, studies are carried out separately, revealing two distinct directions. The behavior of tourist flow is affected by the subjective psychological factor, the subjective cognition and psychological building of tourists constitute the major factors determining their decision-making on flow behaviors (Zahari et al., 2020). The value perceived by tourists in terms of landscape display, infrastructure facilities, traveling costs, and the natural environment imposes a direct impact on their willingness to engage in tourism flow (Kim and Thapa, 2018). Such perceived value will further affect the sensory image of tourism destinations and even the willingness of other tourists on a broader scale (Chen et al., 2021). The behavior of tourist flow is also affected by the objective psychological factor, such as geographical surrounding, spatial distance, and cultural connotation of tourism destinations, which will impose an impact on the decision-making by tourist behavior (Li and Katsumata, 2020), can lead to the flow of tourists. In general, both sorts of studies are based on scientific reasoning. Although seemingly the two factors do not exist in the same space and time, the causal relationship is significant in this chain of connection: objective factors for destination → behaviors of tourist flow → subjective factors for meeting demand,” which ought to be interpreted in the same theoretical framework. Therefore, this study put forward the point-line theoretical framework in the space and time of tourism destinations and attempts to regard the behavior of tourist flow as an intermediary between tourists and destinations, thereby forming research literature that considers the development law of both subjective and objective factors related with tourism destinations. In this study, we put forward the Hypotheses *H2* and *H3*:

*H2: The optimization of tour routes can impose a positive impact on the generation of the behavior of tourist flow.*

*H3: The emergence of the behavior of tourist flow can impose a positive impact on the satisfaction of tourists’ demands in the psychological perception.*

The research on the behavior of tourist flow has vital implications. In particular, the research on constructing the trajectory of space and time is of great significance to the marketing and management of tourism destinations, holds the key to enhancing environmental and exhibition design. It can improve the forecast of tourist flow as well as the distribution and allocation of such flow. Therefore, scholars have carried out studies on the behavior of tourist flow from numerous aspects. For instance, Li and Katsumata (2020) report the mutual correlation among the behaviors of decision-making on entrance transfer by tourists in inbound tourism, the time of entry at the crowded entrance and the time saved at the diversion entrance, and elaborated on the design rules of the schemes of controlling tourist flow. Du et al. (2020) study tourist flow driven by film broadcast, elaborated on the regularity from the perspectives of total tourism volume, the structure of tourism flow network as well as the mode of space and time of tourists, thereby identifying the mechanism of the behavior of tourist flow in the film-induced tourism. Caldeira and Kastenholz (2020) studied the regularity of tourist behaviors in varying spaces and times, elaborated on the necessity and feasibility of tracing tourist activities in a precise manner, and concluded on the vital implications of valuing the behavior of tourist flow in tourism to urban destinations. The extant literature examined the role of tourist flow during resource allocation by leveraging the data on digital footprint collected in the tourism diaries available online and concluded on the significance imposed by the attenuation of distance and the popularity of scenic areas on the spatial pattern of tourism flow, the uneven distribution of core nodes of tourism, the existence of structural holes, and the forming mechanism of core areas of tourism. Numerous studies have been carried out on the behavior of tourist flow, which contains in-depth explanations on the significance of research on such behavior. Based on the insights into the regularity behind, scientific policy recommendations can be provided for regional planning at the macro level. Based on the relevant research on tourist psychological perception, tour routes, and the behavior of tourist flow, it is found that the behavior of tourist flow could potentially connect the tourist psychological perception with tour routes. Therefore, in this study, we put forward the Hypothesis *H4*:

*H4: The behavior of tourist flow serves as the intermediary variable between tourist psychological perception and tour routes.*

## Research Design

To further integrate social communication into transportation routes, this study selected Jiangxi Province as the case for empirical analysis, which is rich in tourist resources. We are motivated to highlight the cultural connections between tourist psychological perception, travelers’ physical flow, and the traveling destination, using a new perspective and paradigm to uncover the timing, spatial, psychological, cultural evolving process, and social communication significance.

### Case Selection

Tourism destinations located in Jiangxi Province of China are selected for empirical analysis. Jiangxi Province has rich tourism resources and short-distance traveling to neighboring regions, thus, it is a place with huge potential for growth in the tourism industry. In total, there are 11 scenic areas with 5A grade recognized by the Ministry of Culture and Tourism of The People’s Republic of China, including Dajue Mountain Scenic Area in Zixi, Fuzhou City, Jiangwan Scenic Area in Shangrao City, Mingyue Mountain Scenic Area in Yichun City, Tengwang Pavilion Scenic Area in Nanchang City, Wugong Mountain Scenic Area in Pingxiang City, Mount. Gui Scenic Area in Shangrao City, Expo Area of Ancient Kiln and Folk Cultures in Jingdezhen City, Lushan Mountain Scenic Area in Jiujiang City, Jinggang Mount Scenic Area in Ji’an City, Longhu Mount Scenic Area in Yingtan City, Sanqingshan Mount Scenic Area in Shangrao City. Specifically, these 11 scenic areas consist of natural and cultural landscapes, which offer an optimal choice for tourists. Moreover, Jiangxi Province has built convenient transportation networks of highway and railway in all scenic areas, facilitating a large influx of tourists. However, the provincial authorities have neither clearly planned the routes for tourism guidance, nor have they set up a provincial platform for big data incurring in tourism. Given this deficiency, this study attempts to verify the connection between tourist psychological perception and tour transportation in the tourism destinations located in Jiangxi Province. With the use of quantitative data of tourist psychological perception, this study focuses on the internal relationship between tourist psychological perception and tour routes and elaborated on the points and lines in the space and time of tourism destinations. The research findings are expected to free the Jiangxi Province from the shackles constraining the local tourism development, close the loopholes due to the absence of scientific guidance, and facilitate the growth driven by the regional tourism economy. Eventually, the study can lay a theoretical foundation for the point-line logic in the space and time of tourism destinations.

### Research Findings

This study included two parts. The first part focused on empirical analysis of the point-line connection and the second part analyzed the mechanism through the logic of the point-line connection. Part one aims to verify the research hypotheses specified in the previous section, to clarify and validate the relationship existing between tourist psychological perception and tour routes in the 11 tourism destinations located in Jiangxi Province, and to explain the point-line interaction in the space and time perspectives, which is based on the observation of element composition. To achieve the stated goals, five tourist groups from the same region with the same age (between 30 and 40 years old), from August 2020 to June 2021 have been surveyed (refer to Table 1).

**TABLE 1:** The demographic statistics of samples (*n* = 112).

Demographics	Category	Distribution	Percentage
Gender	Male	53	47.33%
	Female	59	52.67%
Education	Non-Bachelor	74	66.07%
	Undergraduate	26	23.21%
	Graduate	12	10.72%
Occupation	Enterprise	27	24.11%
	Government	49	43.75%
	Freelance	22	19.64%
	Other	14	12.50%
Monthly Income	<¥5000	9	8.04%
	¥5001-6000	21	18.75%
	¥6001-7000	17	15.18%
	¥7001-8000	23	20.53%
	>¥8000	42	37.50%

Part two served as an extension of Part one, which provided a content analysis based on the regularity of the point-line interaction in the space and time perspectives, and this part included six steps. First, keywords of “5A scenic areas in Jiangxi Province” and “tour transportation” were searched at Chinese biggest social media platform of *Weibo* resulting in a total of 97,648 online traveling posts on the 5A Scenic Areas in the online data extracted from January 1, 2021, to June 30, 2021. Second, keywords were summarized and sorted out by the frequency of these words based on the analysis of the collected online traveling posts. Third, we identified the emotional bias among the online traveling posts with the adoption of semantic analysis logic, which sorts out the regularity of expression of positive, neutral, and negative emotions. Fourth, we illustrate the diagrams of mentioned frequency and emotional distribution based on online traveling posts with the analytical software of Arcgis10.2 (Esri, Inc., United States) and holistically sort out the categorization of tourist psychological perception. Fifth, traffic lines of both railway and highway located in Jiangxi Province were proposed to illustrate a diagram of comparative observation on the tourist psychological perception and tour routes. Finally, we provide policy suggestions on the mechanism and thinking on the point-line connections in the space and time of tourism destinations, which located in Jiangxi Province. Therefore, based on the above observation, we proposed theoretical concepts which have practical implications driven by sound research findings.

## Study 1: Empirical Analysis on the Relationship of Point-Line Connection

Based on the literature review and proposed research hypotheses, the relationship of point-line interaction exists between tourist psychological perception and tour routes, and the behavior of tourist flow serves as the intermediary connecting the two factors. Therefore, the relational models of *H1, H2, H3, and H4* were preliminarily established.

### Model Development and Variable Selection

**FIGURE 1:**
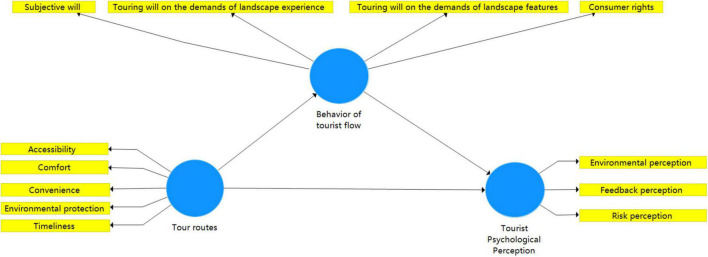
The point and line linking model of tourism destinations in the space and time dimension.

To verify the potential relationship among the three variables, the corresponding scales are introduced to measure the structural model. Specifically, in terms of measuring the tour transportation, the scale of evaluating traffic quality proposed by Compares (Konda and Nakamura, 2020) and the scale of evaluating traffic experience quality proposed are introduced to establish five indicators of measuring the convenience, comfortableness, environmental protection, accessibility, and timeliness of tour transportation through expert assessment and verification. Regarding measuring the behaviors of tourist flow, the destination brand experience scale (DBE) is adopted including a subjective will, touring will on the demands of landscape features, touring will on the demands of landscape experience, and consumer rights as the 5 indicators of measuring the behavior of tourist flow (Zeng and He, 2019). Regarding the measurement of the tourist psychological perception, the risk perception scale and satisfaction measurement scale on tourist perception (Pellicer and Camprubi, 2021) are adopted to set up three measurement indicators, which are environmental perception, risk perception, and feedback perception. Based on the above dimensions and hypotheses, this study proposed the following research model (refer to Figure 1).

### Reliability, Validity, and Descriptive Statistical Analysis

To ensure the reliability, consistency, and stability of the model and verify whether the experimental data could reflect the stability and consistency of the answers provided by respondents, we have adopted the internal consistency, a reliability index, to carry out the reliability test on the twelve questions of the recovered statistics on pre-survey questionnaire scale. Coefficient α at a value of.922 and Cronbach’s alpha (a reliability index) at the value of 0.922 indicated that the questionnaire features optimal reliability and value for further research. Subsequently, 120 questionnaires were issued with 112 valid questionnaires recovered later, and the effective rate of recovery is 93.3%. For the principal component analysis (PCA) method we used, the valid questionnaires can explain 72.183% of the information. Subsequently, we have conducted the KMO and Bartlett tests using SPSS software (SPSS, Inc., United States). The KMO, a coefficient of the structural validity test, amounts to.895, exceeding.8 thresholds, and the level of significant amounts to 0, less than.05, indicating optimal validity and a high level of data fitting (refer to Table 2). The analysis of the structural equation model was carried out to examine the rationality of the original model development and research hypotheses.

**TABLE 2:** Validity test.

KMO and Bartlett tests		
Kaiser-Meyer-Olkin		0.895
Bartlett Test of Sphericity	Approximate chi-square	1076.59
	Free degree	66
	Conspicuousness	0

The descriptive statistical analysis was also carried out on the varying dimensions of tour routes, behaviors of tourist flow, and tourist psychological perception. The effective sample data following the normal distribution, we measured both the mean and SD of the questions for descriptive analysis. Specifically, the mean and SD of tour routes include convenience (3.85, 0.851), comfort (3.84, 0.823), environmental protection (3.83, 0.889), smoothness (3.98, 0.816), and timeliness (4.05, 0.837). The mean and SD of the behavior of tourist flow include subjective will (4.11, 0.798), touring will on the demands of landscape features (4.16, 0.742), touring will on the demands of landscape experience (4.08, 0.829), and consumer’s rights (4.15, 0.785). The mean and SD of tourist psychological perception includes environmental perception (4.18, 0.762), risk perception (4.02, 0.747), and feedback perception (3.96, 0.821). The quantitative indicators fall within the acceptable range of the measurement standards (refer to Table 3). Therefore, the recovered data were eligible for further verification through the statistical analysis in the next step.

**TABLE 3:** Descriptive statistics.

Item	Mean	STDEV	Effective cases	Missing cases
Convenience	3.85	0.851	112	0
Amenity	3.84	0.823	112	0
Environmental	3.83	0.889	112	0
Smooth	3.98	0.816	112	0
Timeliness	4.05	0.837	112	0
Subjective will	4.11	0.798	112	0
Scenic Area features	4.16	0.742	112	0
Scenic Area experience	4.08	0.829	112	0
Consumer’s right	4.15	0.785	112	0
Environmental perception	4.18	0.762	112	0
Risk perception	4.02	0.747	112	0
Feedback perception	3.96	0.821	112	0

### Test of Reliability and Validity of the Structural Equation Model

Based on the general requirements of the samples and the number of variables, the approach of partial least squares (PLS) is adopted as the research method in this study, which is applicable for the small sample analysis and capable of processing the non-normal distribution data. In the 1970s, the Swedish econometrician Wold (1975) proposed a partial least-squares regression method, which later evolved to partial least squares structural equation modeling (Mateos-Aparicio, 2011). Partial least squares structural equation modeling (PLS-SEM) estimates the parameters of a set of equations in a structural equation model by combining principal component analysis with regression-based path analysis (Hair et al., 2012). Since the PLS-SEM method can effectively overcome the collinearity between observed variables, eliminate the effect imposed by the noise that is inconducive to regression, and provide the PLS-SEM model with good robustness (Sarstedt et al., 2020), PLS-SEM is adopted as an effective tool in this study.

**FIGURE 2:**
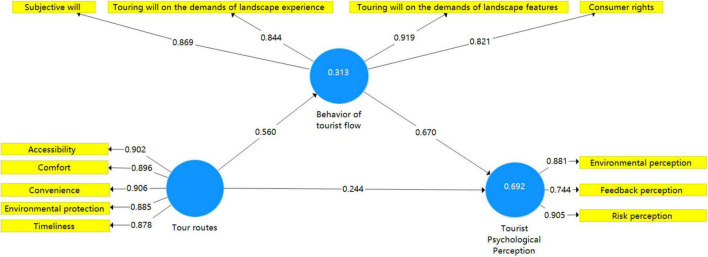
Reflective measurement model.

Specifically, a single-dimensional examination is carried out on the second-level indicators of three first-level indicators. Based on the analysis results, the eigenvalue of the first principal component of each dimension is found to be greater than 1 and the rest of the eigenvalues are smaller than 1. Moreover, the results indicate that each dimension has passed the examination. Subsequently, based on the reflective measurement, the model validation of the point-line relationship in the space and time of tourism destinations (refer to Figure 2) is set up with the use of the PLS algorithm, and the software of Smart PLS 3.3 (SmartPLS GmbH, Germany) was applied. Based on the analysis results, the normed fit index (NFI) amounts to.846, which is consistent with the requirements of model fitting.

The validation results on tour routes, tourist psychological perception and behavior of tourist flow include Cronbach’s alpha (0.937, 0.802, 0.886), rho_A (0.939, 0.85, 0.889), CR (0.952, 0.883, 0.922) and AVE (0.798, 0.716, 0.746). All the index coefficients are greater than the threshold of.7, indicating that the model features optimal reliability. The R^2^ amounts to.692, indicating that the exogenous variables could impose a significant impact on the endogenous variables and the model features a strong correlation predictive capability. The Q^2^ amounts to.47, indicating that the external variables feature a strong correlation predictive capability for the endogenous variables and the model features a strong predictive capability overall. In addition, the diagonal of the matrix, which is the square root of the average variance extracted (AVE) of each latent variable, is greater than.5 and each latent variable has varying connotations and features an optimal discriminant validity in theory (refer to Table 4). Based on the significance test of Bootstrapping path coefficient, the T values of the variables are specified as follows: tour routes→tourist psychological perception (2.099), tour routes→behavior of tourist flow (4.593), the behavior of tourist flow→tourist psychological perception (5.938), tour routes→behavior of tourist flow→tourist psychological perception (5.782) according to the specific indirect effect test. All these variables feature high statistical *T* values, indicating that each of the path coefficients has passed the significance test and the model structure features good stability (refer to Table 5).

**TABLE 4:** Results of model reliability validity test and fitting index.

Indicator	Cronbach’s Alpha	Rho_A	CR	AVE	R^2^	Q^2^
Tour Routes	0.937	0.939	0.952	0.798	0.692	0.47
Tourist Psychological Perception	0.802	0.85	0.883	0.716		
Behavior of tourist flow	0.886	0.889	0.922	0.746		
						

**TABLE 5:** Results of the significance test of the path coefficients.

Indicator	O^2^	M	STDEV	*T*	P
Tour Routes - > Tourist psychological perception	0.244	0.276	0.116	2.099	0.036
Tourism and transportation routes - > Behavior of tourist flow	0.56	0.576	0.122	4.593	0
Behavior of tourist flow - > Tourist psychological Perception	0.67	0.642	0.113	5.938	0
Tour Routes - > Behavior of tourist flow - > Tourist psychological perception	0.375	0.36	0.065	5.782	0

### Discussion of the Path Coefficients of the Structural Equation Model

Based on the above research findings and the previous theoretical derivation, the path coefficients of the structural equation model can be explained as follows: The deployment of tour routes can impose a positive impact on the tourist psychological perception, but the two factors have not shown a strong relationship, thus, verifying *H1*: The optimization of tour routes can impose a positive impact on the improvement of tourist psychological perception. It shall be noted that the convenient access to transportation of renowned tourism destinations is one of the factors affecting the overall perception of tourists but not a vital factor. Such a relationship could explain why certain tourist attractions located in remote areas can draw tourists in an endless stream. In case of a perfect layout of tour routes, it can significantly enhance the level of satisfaction of tourists as well as their overall perception and experience of the target tourism destinations.

In the tourism environment, the layout of tour routes can play a vital role in attracting the behavior of tourist flow, thus, verifying *H2*: The optimization of tour routes can impose a positive impact on the generation of the behavior of tourist flow. Such regularity has revealed the correlation between tourism conditions and tourist behavior. In other words, under numerous circumstances, tourists are only able to follow the existing tour routes initially designed in the tourism destinations due to the fear of unknown areas despite their desire to explore new tour routes. This phenomenon has fully validated the value of the physical conditions of tour routes.

Behavior serves as the forerunner of psychological mapping while the behavior of tourist flow could reflect the changes taking place in their perceptual psychology, thus, verifying *H3*: The emergence of the behavior of tourist flow can impose a positive impact on the satisfaction of tourists’ demands in the psychological perception. Just as the empirical study has concluded, many practices show that properly guiding the behavior of tourist flow can effectively enhance their perception and experience during landscape touring. In other words, walking into the tourism destination from an entrance with a luxuriant growth of flowers and vegetation is far more agreeable than entering the destination from a barren and dilapidated place, which can better satisfy tourists’ demands on psychological perception.

Proper layout of tour routes can provide high-quality guidance and even promote the generation of the behavior of tourist flow. Moreover, it can enhance the overall experience of tourists while satisfying their psychological demands, thus verifying *H4*. Judging from this research finding, given that the behavior of tourist flow serves as a connection of points and lines in the space and time of tourism destinations, effectively monitoring such behavior helps predict the changing trend of tourist psychological perception and explain the practical issues faced by tour transportation. Therefore, further studies need to be carried out to draw deeper insights on how to enhance controlling behavior of tourist flow, in addition to the content analysis on the logic of the point-line connection.

## Study 2: Content Analysis of the Point-Line Connection Logic

This study is based on the content analysis of 97,648 effective traveler’s online posts after crawling and cleaning, the software of Arcgis 10.2 is adopted in the procedure of visualization process, to obtain the mentioned frequency in 11 national 5A scenic areas located in Jiangxi Province in the texts of random traveler’s online posts.

### Layout and Features of Tourist Psychological Perception

**FIGURE 3:**
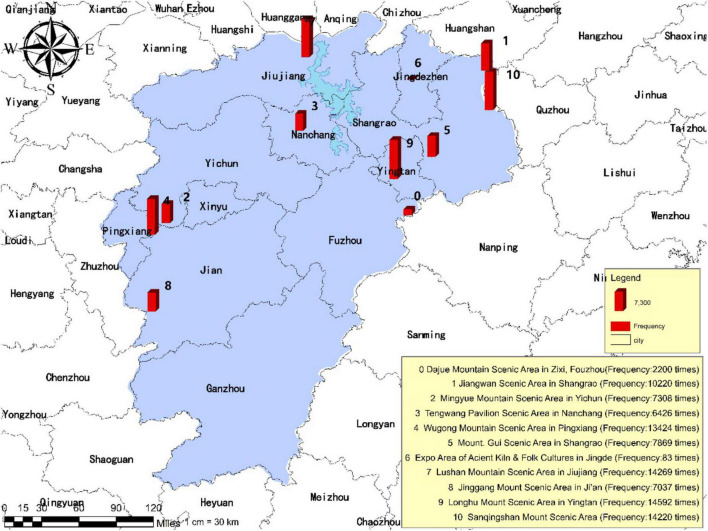
Frequency of tourism destinations in tourist’s psychological perception.

**FIGURE 4:**
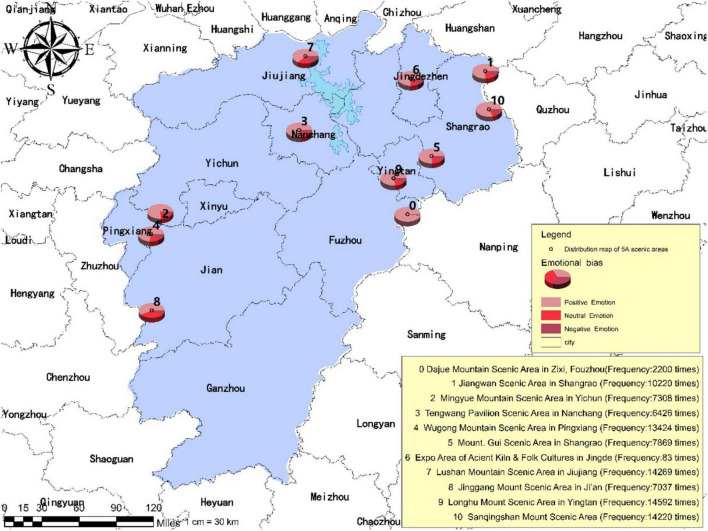
The emotional distribution of tourism destinations in tourist’s psychological perception online posts.

As illustrated in Figures 3, 4, the frequency of mentioning is significantly higher for the Longhu Mount Scenic Area located in Yingtan City, the Sanqingshan Mount Scenic Area located in Shangrao City, and the Lushan Mountain Scenic Area located in Jiujiang City compared with other scenic areas such as the Jinggang Mountain Scenic Area located in Ji’an City, the Wugong Mountain Scenic Area located in Pingxiang City, the Expo Area of Ancient Kiln and Folk Cultures located in Jingdezhen City and the Mingyue Mountain Scenic Area located in Yichun City, occupying a larger share of the traveler’s online posts published by tourists. The results of the mentioned frequency have revealed the tourist psychological perception of each tourism destination.

Based on the commonality analysis of the scenic areas with the top-ranking mentioned frequency, it is found that tourists tend to prefer the scenic areas of natural landscapes with cultural ambiance rather than the scenic areas of purely cultural and historical landscapes as well as the commercial landmarks. The results indicate that the interest orientation of tourists in contemporary days has experienced the transition from target-oriented tourism into demand-oriented tourism. The preference of staying away from the hustle and bustle of the city and appreciating the mountain scenery has been gradually formed in certain tourist groups, thus inhibiting individual desire for scenic areas of purely cultural and historical landscapes, and strengthening their fonds with scenic areas of natural landscapes. On the other hand, convenient access to transportation also constitutes one of the vital factors considered of tourists’ provincial tourism. As can be seen from the Geographic Information System (GIS) map, the mentioned frequency of national 5A scenic areas located in northern Jiangxi Province proves to be significantly higher than that in southern Jiangxi Province. This is because the transportation infrastructure in northern Jiangxi is superior to that in southern Jiangxi, like Nanchang City, i.e., the capital of Jiangxi Province which is in northern Jiangxi. The convenient access to transportation determines the planning of tour routes and imposes an impact on the perception and experience of tourists during in-depth tourism. Therefore, the building of transportation facilities plays a critical role in the development of provincial tourism.

### Layout and Status of Tour Routes

**FIGURE 5:**
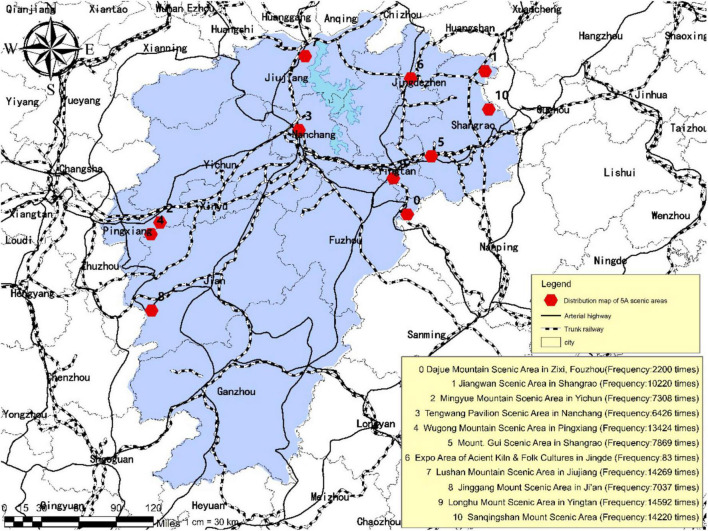
The tourism routes distribution of tourism destinations in Jiangxi Province.

Judging from the *status quo* of national 5A scenic areas and provincial traffic conditions in Jiangxi Province (refer to Figure 5), it can be noted that the transportation infrastructure in northern Jiangxi Province is superior to that in southern Jiangxi Province. In addition, Jiangxi has rich resources of natural landscapes, thus producing an inclination that tourists prefer the northern part to the southern part. The scenic areas of natural landscapes that tourists prefer are distributed in the northeastern and southwestern parts of Jiangxi Province. In the north of Jiangxi, the traffic network is high in density and the scenic areas are close to each other, the region has convenient access to both railway and highway transportations. By contrast, in the south of Jiangxi, the traffic network is sparse, and the railway proves to be a more comfortable means of transportation than road traffic. Tourists tend to have varying psychological perceptions for different scenic areas and the ups and downs of such perceptions can make up for the emotional gap brought by varying scenic areas, thus enabling tourists to strike a balance between the cultural landscape and the natural landscape.

### The Mechanism of Point-Line Connection

**FIGURE 6:**
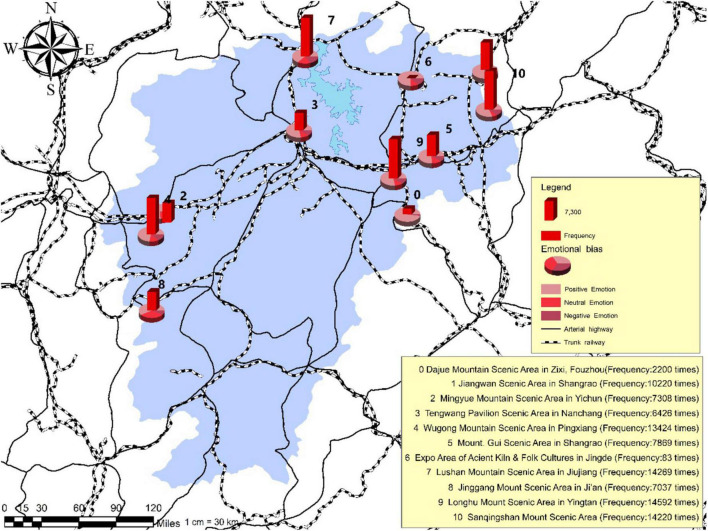
The trunk railway points and line links of tourism destinations in the space and time dimension.

**FIGURE 7:**
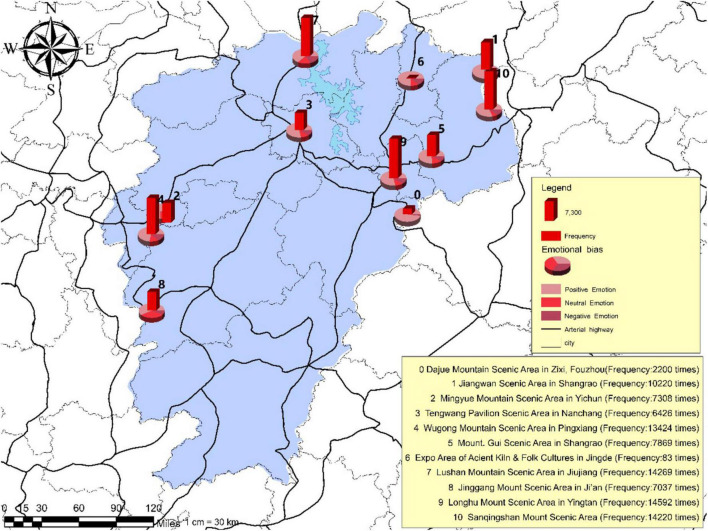
The arterial highway point and line links of tourism destinations in the space and time dimension.

Based on the study of the features, distributions of tourist psychological perception as well as the layout and *status quo* of tour routes, the tourist psychological perception is found to have a significant correlation with the layout of tour routes. Besides the Wugong Mountain Scenic Area, located in Pingxiang City, is not on the highway or railway lines, the Longhu Mountain Scenic Area located in Yingtan City and the Sanqing Mountain Scenic Area located in Shangrao City, are not on the railway lines; other scenic areas in Jiangxi are all located on the major traffic lines. Despite the mountainous areas where tour routes are hard to be operated, the planning of tourism destinations in Jiangxi Province has revealed a high correlation between the tourist psychological perception and tour routes. From the perspective of transportation network distribution, the distribution of railway lines is far denser in northern Jiangxi Province than in the southern part of the province and the distribution of arterial highway lines in northern Jiangxi Province is also relatively denser than that in the southern part of the province (refer to Figures 6, 7). Therefore, the logic of the point-line connection in the space and time of tourism destinations in Jiangxi Province is once again verified in practice, whereas its internal characteristics would require more in-depth study.

Specifically, among the 97,648 pieces of UGC captured in this study, there are 2,200 items on the Dajue Mountain Scenic Area located in Zixi County, Fuzhou City; 10,220 items on the Jiangwan Scenic Area located in Shangrao City; 7,308 items on the Mingyue Mountain Scenic Area located in Yichun City; 6,426 items on the Tengwang Pavilion Scenic Area located in Nanchang City; 13,424 items on the Wugong Mountain Scenic Area located in Pingxiang City; 7,869 items on the Guifeng Scenic Area located in Shangrao City; 83 items on the Expo Area of Ancient Kiln and Folk Customs Cultures located in Jingdezhen City; 14,269 items on the Lushan Mountain Scenic Area located in Jiujiang City; 7,037 items on the Jinggang Mountain Scenic Area located in Ji’an City; 14,592 items on the Longhu Mountain Scenic Area located in Yingtan City; 14,220 items on the Sanqing Mountain Scenic Area located in Shangrao City. Based on the preliminary analysis, among all data, 70,749 items revealed positive emotions (72.08%), 16,160 items revealed neutral emotions (16.46%), and 11,239 items revealed negative emotions (11.45%) (refer to Table 6). The research findings indicate that the tourism resources in Jiangxi Province have drawn extensive interest from tourists and in turn, the tourists have optimal experience feedback overall. However, there are still negative emotions that need to be alleviated through the improvement by custom-tailored schemes. It is also verified that the texts of 97,648 online traveling posts have significant features of emotion carrier and can serve as scientific and effective data for further analysis.

**TABLE 6:** The mechanism of point and line connections.

Code	Scenic areas	Frequency	Positive emotion	Neutral emotion	Negative emotion
0	Dajue Mountain Scenic Area in Zixi, Fouzhou	2200	2150	0	50
1	Jiangwan Scenic Area in Shangrao	10220	8066	1638	1016
2	Mingyue Mountain Scenic Area in Yichun	7308	5528	1142	638
3	Tengwang Pavilion Scenic Area in Nanchang	6426	5012	794	620
4	Wugong Mountain Scenic Area in Pingxiang	13424	9735	1944	1745
5	Mount. Gui Scenic Area in Shangrao	7869	5988	1104	777
6	Expo Area of Acient Kiln & Folk Cultures in Jingde	83	62	10	11
7	Lushan Mountain Scenic Area in Jiujiang	14269	8954	2906	2409
8	Jinggang Mount Scenic Area in Ji’an	7037	4063	2472	502
9	Longhu Mount Scenic Area in Yingtan	14592	11369	1681	1542
10	Sanqingshan Mount Scenic Area	14220	11500	1020	1700

Based on the datasets of cognitive vocabulary and word mentioned frequency, an analytical diagram on the semantic network of national 5A grade scenic areas in Jiangxi Province is established with the keywords of 5A scenic areas in Jiangxi Province and transportation, looking at the potential connection of the high-frequency words mentioned in the texts, tourists tend to focus on the city where the scenic area is located as well as the name and features of the scenic area, revealing the focus of tourists’ assessment on tour transportation in Jiangxi Province. Specifically, the city where the scenic area is located will determine how tourists arrange their accommodation, traveling, and means of transportation, thereby forming a circle of tourism radiation with the city as the core. All the scenic areas located in the circle will become the preferential options for tourists, thereby forming a differentiated model of tour transportation and leading to the transport radiation-oriented tourism behaviors. In addition, from the outset of traveling, tourists generally establish a psychological presupposition for the intended destination to clarify their goals for tourism. Under such circumstances, the tour transportation will drive tourists to generate their traveling preferences, leading to target-oriented tourism. Destination selection is largely guided by perceptual psychology, especially the features of tourism destinations such as folk customs and expositions. Moreover, to design demand-oriented tourism tour transportation can connect the features of scenic areas in a circle-shaped manner. In the nutshell, there are three patterns of point-line interaction in the space and time of tourism destinations, such as transportation radiation-oriented tourism, target-oriented tourism, and demand-oriented tourism. These patterns correspond to three kinds of relationships, which are psychological preference and tour transportation radiation, psychological presupposition and tour transportation planning, and psychological inclination and interaction with the tour transportation circle.

## Conclusion and Discussion

We have conducted two studies to elaborate the relationship of point-line connection in the space and time of tourism destinations located in Jiangxi Province and analyzed the expression of online posts in the logic of point-line connection, and reached the following three main conclusions.

First, tourist flow represents the point-line connection in the space and time of tourism destinations. Previous studies have been carried out on the tourist psychological perception and tour routes, which is generally taken for granted that both factors are directly related despite the lack of logical reasoning. Therefore, this study has applied tourists’ psychological perception as the point and tour routes as the line to elaborate their relationships. Based on our findings, although the point and the line do not exist in the same physical space, while there is a connection between the two factors. Specifically, tourist flow is initiated from the tourist psychological perception and the optimization of tour routes can impose a positive impact on the tourist psychological perception as well as the behavior of tourist flow. Furthermore, tourist flow can impose a positive impact on the satisfaction of tourists’ demands of psychological perception and in turn, serves as the intermediary variable between tourist psychological perception and tour routes.

Second, three patterns of point-line interaction modes have been observed in the space and time of tourism destinations, such as psychological preference and tour transportation radiation, psychological presupposition and tour transportation planning, and psychological inclination and interaction with tour transportation circle. The findings of this research are derived from the GIS observation and the analysis of the online traveling textual posts, which have integrated the subjective understanding with the objective perception of the image of tourism destinations. Moreover, the study has further empirically examined the role of tourist flow in connecting tourist psychological perception and tour routes and presented the logic of the point-line connection in the space and time of tourism destinations located in Jiangxi Province.

Third, through the case analysis, the study will enable local policymakers to build local tour transportation. Specifically, tourists tend to focus on the city where the scenic area is located as well as the name and features of the scenic area, which reflects the overall image, objective, and demand of tourists. The finding of this research has specified the psychological dimension of tourists’ perception of the tourism destinations located in Jiangxi Province and pointed out the future orientation for the publicity of tourism destinations. In addition, the practical features of the tour routes located in Jiangxi Province are clarified in this study. Due to the features of mountainous areas, the means of transport in Jiangxi Province is primarily railways and highways, and thus, the connection between tourism destinations depends on such means of transportation. Judging from the GIS observation, the distribution of railways and highways in northern Jiangxi Province is denser than that in the southern part of the province, depending on the impact of scenic areas or the distribution of cities to a certain extent. Therefore, the planning and optimizing of tour routes shall focus on the effective connection among scenic areas, which is based on the extension and expansion of existing tour routes. The features of tourist flow in Jiangxi Province are primarily the connection between objectives and demands. Thus, it is necessary to monitor tourist flow and identify its regularity timelier and more accurately for future planning of tour transportation, and further enhance the system of tour transportation in Jiangxi Province.

## Data Availability Statement

The original contributions presented in the study are included in the article/supplementary material, further inquiries can be directed to the corresponding author/s.

## Ethics Statement

Ethical review and approval were not required for the study on human participants in accordance with the local legislation and institutional requirements. The patients/participants provided their written informed consent to participate in this study.

## Author Contributions

XW and YL organized the theoretical background and wrote the literature review. LZ and FW performed the statistical analysis and completed the manuscript. YZ and SZ designed and revised the manuscript. All authors contributed to the article and approved the submitted version.

## Conflict of Interest

The authors declare that the research was conducted in the absence of any commercial or financial relationships that could be construed as a potential conflict of interest.

## Publisher’s Note

All claims expressed in this article are solely those of the authors and do not necessarily represent those of their affiliated organizations, or those of the publisher, the editors and the reviewers. Any product that may be evaluated in this article, or claim that may be made by its manufacturer, is not guaranteed or endorsed by the publisher.
